# Supplementary material for: Multiplexed Cas9 targeting reveals genomic location effects and gRNA-based staggered breaks influencing mutation efficiency
Source: Nat Commun. 2019 Apr 8;10:1598. doi: 10.1038/s41467-019-09551-w (PMC6453899; doi:10.1038/s41467-019-09551-w)
Supplement: Supplementary file 1 — Supplementary Information [file 41467_2019_9551_MOESM1_ESM.pdf]

Supplementary Information

Multiplexed Cas9 targeting reveals genomic location effects and gRNA-based staggered breaks influencing mutation efficiency

Santiago Gisler[1*], Joana P. Gonçalves[2,3*], Waseem Akhtar[1*], Johann de Jong[3,4], Alexey V. Pindyurin[5,6], Lodewyk F. A. Wessels[2,3†], Maarten van Lohuizen[1†]

[1] Division of Molecular Genetics, Oncode and The Netherlands Cancer Institute, Amsterdam, The Netherlands
[2] Department of Intelligent Systems, Delft University of Technology, Delft, The Netherlands
[3] Division of Molecular Carcinogenesis, Oncode and The Netherlands Cancer Institute, Amsterdam, The Netherlands
[4] Data & Translational Sciences group, UCB Biosciences GmbH, Monheim am Rhein, Germany
[5] Institute of Molecular and Cellular Biology, Siberian Branch of Russian Academy of Sciences, Novosibirsk, Russia
[6] Division of Gene Regulation, Oncode and The Netherlands Cancer Institute, Amsterdam, The Netherlands

*** These authors contributed equally to this work.**
† Correspondence: l.wessels@nki.nl (L.F.A.W.), m.v.lohuizen@nki.nl (M.v.L.).

Supplementary Note 1 – **Impact of TRIP integrations on correlation between expression and mutation frequency**

The modest correlation between IR mutation frequency and IR expression (Figures 3B-3D and Supplementary Figure 3), measured in TRIP cells with the same TGE landscape, suggests that additional factors might be involved in determining the locus-specific variation in IR mutation frequency. Here, we set out to investigate whether changes eventually caused by the integration of TRIP reporter genes could explain the weak association between Cas9-induced mutation frequency and expression observed in our study.

We analyzed endogenous expression quantified in wild-type mES cells (WT expression), and also expression measured in our multi-promoter TRIP mES cells (IR expression). Both WT and IR expression levels correlated well with publicly available wild-type TGE features, which was reassuring (Figure 3D and Supplementary Figure 1). The correlation was higher for IR expression than WT expression, possibly also due to a difference in quantification methods: WT expression was determined using a standard approach based on RNA-seq counts alone for a region of 2kb around the integration site, while IR expression resulted from cDNA counts normalized by corresponding genomic DNA counts for a region of 168bp spanning the Cas9 target sites (following the TRIP protocol).

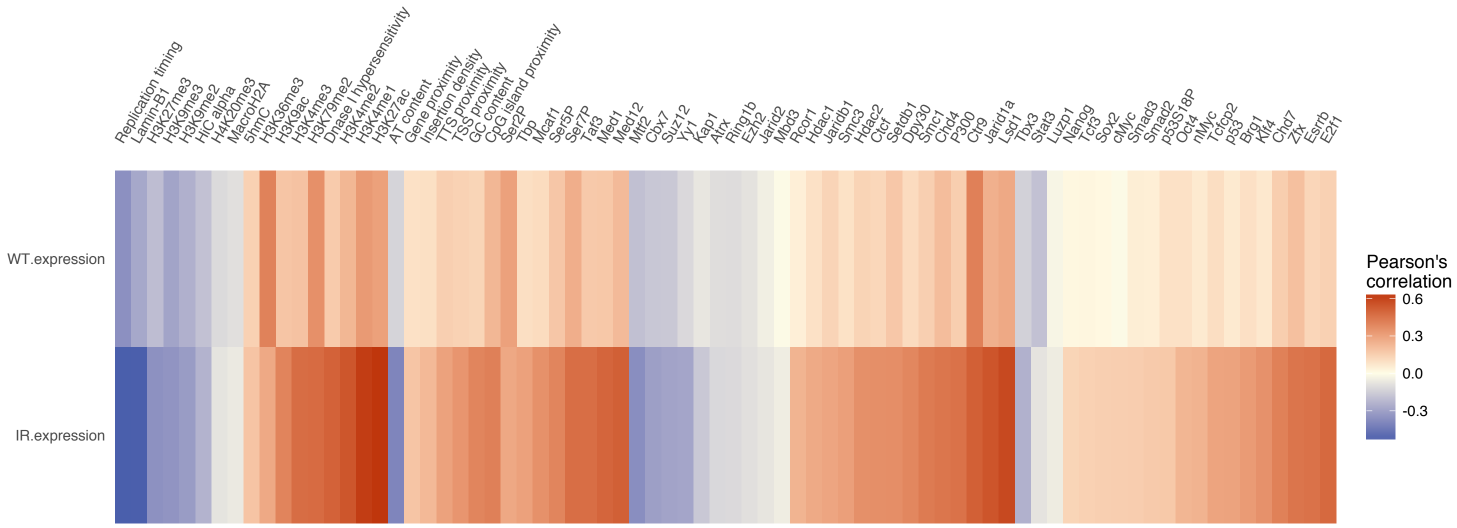

Supplemetary Figure 1: Correlation of expression levels at IR loci in mES TRIP cells (IR expression) and at the corresponding endogenous loci in wild-type mES cells (WT expression), with TGE features of wild-type mES cells. Note: “IR expression” is the same as “IR expression” or “Reporter expression” in Figure 3D. “WT expression” is the same as “Gene expression” in Figure 3D.

To exclude larger variations in expression due to the integration of exogenous DNA and/or quantification methods, we selected two groups of integration loci showing consistent high or low expression both in wild-type (WT) mES cells and in our mES TRIP pool (IR). We defined high and low expression respectively as values in the top and bottom 25 percentiles of the expression distributions. The result of this selection based on the WT and IR expression value distributions is depicted in Supplementary Figure 2. This figure shows some variability in IR (or WT) expression when selecting the IR groups based on high/low WT (or IR) expression only, but a very clear separation between the expression distributions of the high/low IR groups based on both WT and IR expression.

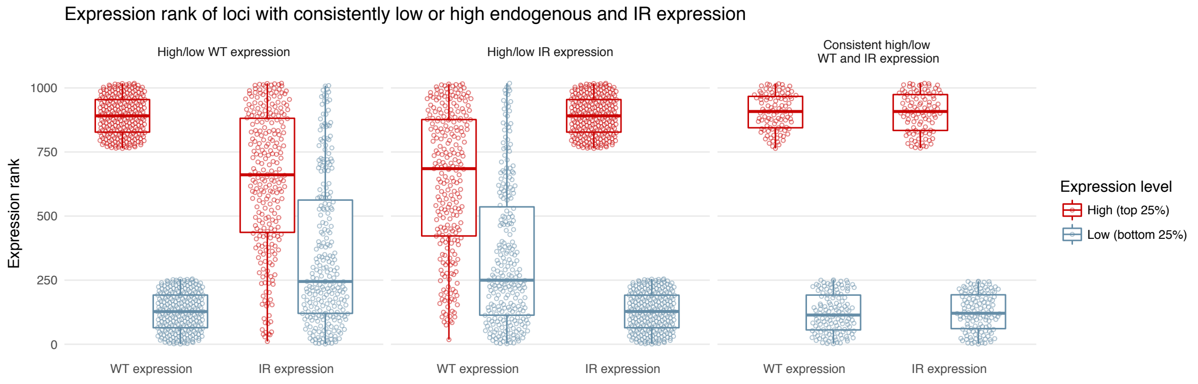

Supplementary Figure 2: Distributions of WT and IR expression levels for groups of loci with (left) high/low WT expression, (middle) high/low IR expression, and (right) consistent high/low WT and IR expression. Each circle corresponds to a distinct IR locus. Red and blue denote groups of loci with high (top 25%) and low (bottom 25%) expression, respectively.

Assuming that the correlations we observed between expression and Cas9-induced mutation frequency would have been heavily impacted by the integration of exogenous DNA, our selection should result in an increase in the difference between IR mutation frequencies across the groups of IRs with consistently high/low WT and IR expression levels, relative to the groups selected based only on WT or IR expression.

Supplementary Figure 3 shows that we could not validate these observations, the selection based on consistent WT and IR expression did not improve the association between mutation frequency and IR expression reported in the main manuscript (Figure 3D). These results show that IRs with similar expression levels exhibited large variation in mutation frequency, and indicate that eventual changes induced by the integration of TRIP reporters were likely not the main source of such variation.

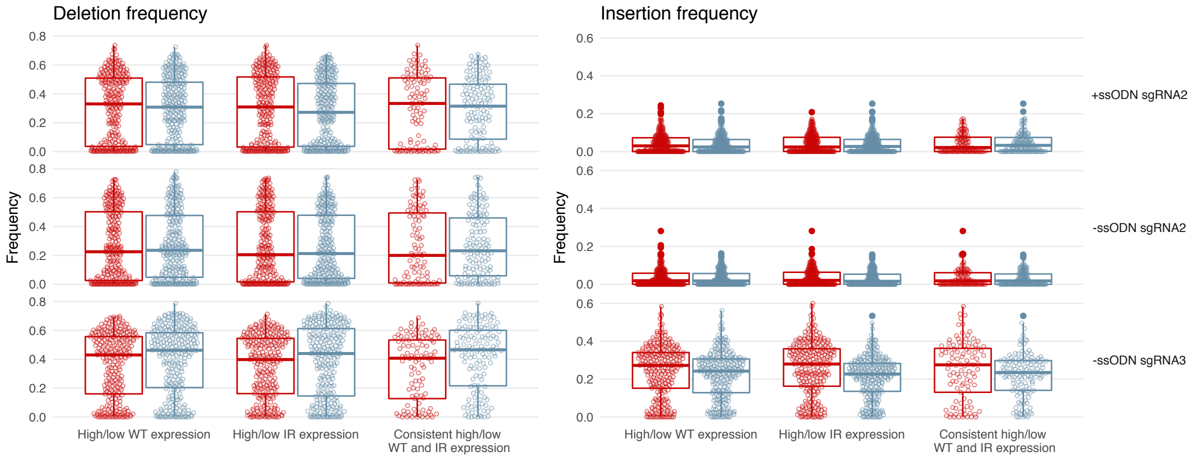

Supplementary Figure 3: Distribution of Cas9-induced mutation frequencies (deletion and insertion) obtained in the three different TRIP pool assays for the same loci groups in Supplementary Figure 2.

***Supplementary Note 2* – Assessing the influence of Cas9 concentration on mutation frequency and patterns**

To assess whether the Cas9 concentration would impact mutation frequency and/or patterns, we analyzed data from assays on 5 populations of cells sorted by different mCherry intensities (samples 1 to 5, with increasing Cas9 concentrations). These were intermediate assays used to fine tune the experimental protocol, and thus did not cover all the conditions of the experiments described in the main manuscript. Specifically, we used a population of mES cells containing varying TRIP integrations with a single promoter, PGK. We targeted the IR loci in samples 1-5 using Cas9 complex. We had lower sequencing depth per IR than we had for the cell line and multi-promoter TRIP assays analyzed in the main manuscript (Supplementary Figure 4).

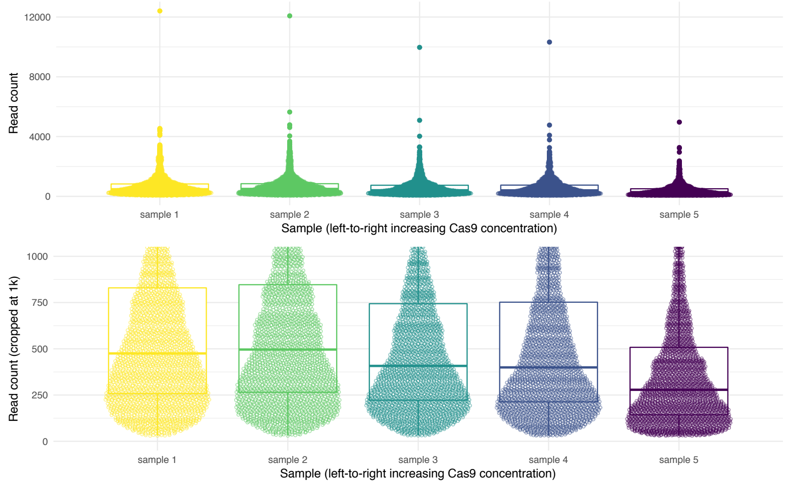

Supplementary Figure 4: Distributions of read counts per IR for five PGK-driven TRIP pools sorted by Cas9 concentration (samples 1 to 5, with increasing Cas9 concentration). Top: entire distribution. Bottom: plot cropped at 1000 read count.

The overall mutation frequency varied significantly with Cas9 concentration, as expected (Supplementary Figure 5, top plot). Despite this, the relative frequencies of the different mutation types remained relatively stable across samples 1-5 (Supplementary Figure 5, bottom plot) and were consistent with the distributions observed for IRs with the PGK promoter from our multi-promoter TRIP pool data (Figure 2C).

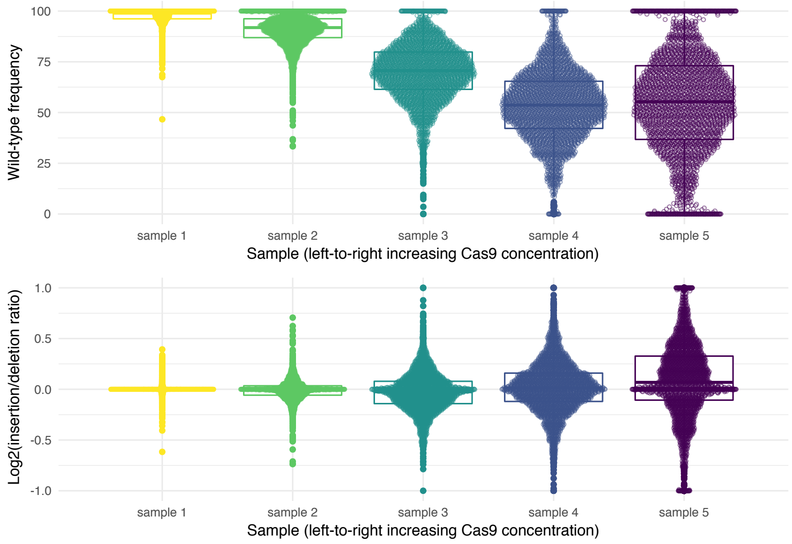

Supplementary Figure 5: Frequency of wild-type reads (top) and log2 ratio between insertion and deletion frequencies (bottom).

Changes in frequencies were very subtle and mostly noticeable using extreme variations in Cas9 concentration (e.g. sample 5 versus the rest). We observed that generally both deletion and insertion frequencies increased with Cas9 concentration. At the highest tested Cas9 concentration (sample 5), insertions became on average slightly more frequent than deletions. In this regard, we note that higher Cas9 concentrations resulted in less sequenced material and lower read counts per IR, possibly due to cell death by toxicity or damage to the DNA caused by the extensive Cas9 cleavage. This could also bias the data towards less damaging mutations (e.g. cells carrying 1-bp insertions could have better survival than those affected by larger deletions).

The 5 samples with different Cas9 concentrations showed similar indel size distributions and patterns (Supplementary Figure 8), corroborating the results in the main manuscript (Figures 4A, 4B, 4D). The most common deletion sizes were {2,4,5}-bp (Supplementary Figure 6), and 1-bp was by far the most frequent insertion size (Supplementary Figure 6), as seen for the TRIP cell line and multi-promoter pools (Figure 4A). The ten most frequent deletions obtained for the samples 1-5 (Supplementary Figure 8, bottom right) also mostly agreed with the results reported for the TRIP cell line (Figure 4B), both in ranking and relative frequency (note that the deleted regions in Supplementary Figure 8 are represented from left to right in decreasing order of frequency according to Figure 4B. Samples 1 and 2 showed more extreme values than the remaining three samples, namely for the frequency of CGTAT (rank 1 in Figure 4B) and TATGCG (rank 7 in Figure 4B). We note that each of these deletions represented a small proportion of the total and that the ratio of mutated reads in these samples was much smaller than in samples 3-5. Therefore, some variability was expected. Finally, the most inserted nucleotide in 1-bp insertions was overwhelmingly T, accounting for over 93% of 1-bp insertions in all 5 samples (Supplementary Figure 8, bottom left). This observation matched the TRIP cell line data as well (Figure 4D).

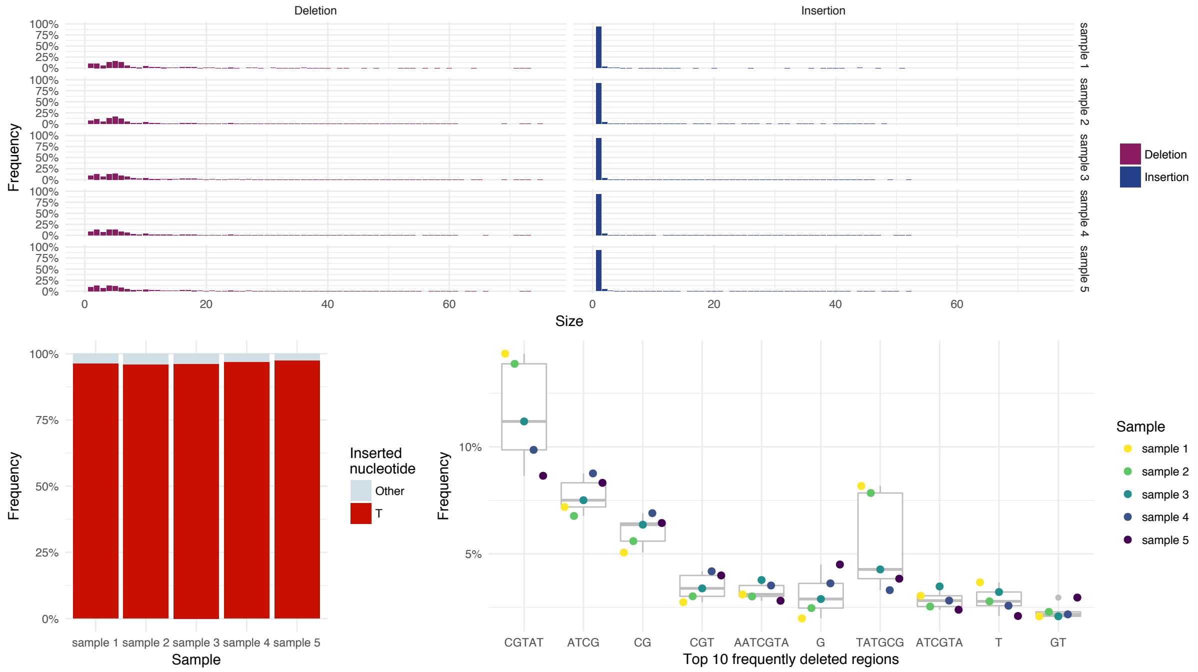

Supplementary Figure 6: Mutation sizes and patterns in five PGK-driven TRIP pools with different Cas9 concentrations. Top left: distribution of deletion sizes in samples 1-5. Top right: distribution of insertion sizes in samples 1-5. Bottom left: frequency of the most inserted nucleotide versus the rest in samples 1-5. Bottom right: frequency of the ten most deleted regions in samples 1-5; the deleted regions are displayed left to right in decreasing order of their corresponding frequency in the cell line (Figure 4B).

Supplementary Note 3 – Frequency of IR-promoter misassociation due to translocations following Cas9 targeting

Our use of RNA-guided Cas9 to target tens of loci per cell could increase the likelihood of long-range translocations, and eventually lead to the association of some IR barcodes with different promoters or TGE features. However, CRISPR/Cas9-induced chromosomal translocation events are rare (Jiang et al., *Sci. Rep.* 2016). Different studies seeking to optimize the frequency of translocations using RNA-guided Cas9 targeting of a pair of loci have achieved efficiencies between 1% and 2% (Lekomtsev et al., *BMC Genomics* 2016; Jiang et al., *Sci. Rep.* 2016). We also note that the loci in these studies are typically involved in tumor-associated translocations and thus could be more likely to translocate than a random pair of loci. Targeting tens of loci per cell could increase the frequency of translocations in general, but such events should remain rather rare for random pairs of loci. In addition, only a fraction of translocation events would lead to the ligation of an upstream to a downstream region of two DSBs. We could not check for this type of translocation, since our sequenced amplicons covered only a region of the IR gene body (EGFP cassette), including the expected DSB site, along with the IR promoter index and the IR barcode downstream, but not the promoter itself located upstream of the IR gene (Supplementary Figure 7). The reason for this was that the complete IR sequence was 1.8-3.4kb in length, which would be challenging to amplify reliably and consistently across ~1k IRs.

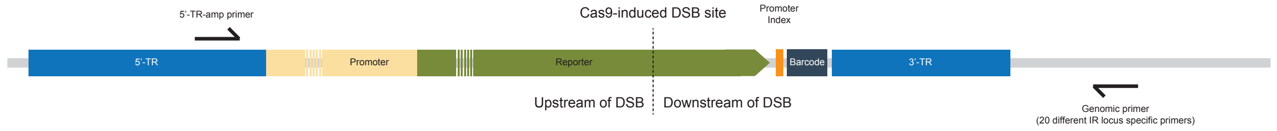

Supplementary Figure 7: Reporter construct and location of amplification primers for assessment of IR-promoter misassociation.

Importantly, our results on mutation frequencies and patterns were largely consistent with other studies targeting only a single to a few loci per cell. Moreover, our estimated IR mutation frequencies correlated strongly across experiments (Figure 2D), which we would not expect if each TRIP mES cell population had suffered a significant number of translocations following Cas9 targeting.

As an additional check, we investigated the occurrence of translocation events for 20 randomly chosen IRs using PCR amplification and Sanger sequencing. For this purpose, we sought to obtain longer amplicons spanning the IR-specific elements upstream and downstream of the expected Cas9 target site (Supplementary Figure 7). The promoter sequence was the most proximal IR-specific feature located upstream of the EGFP cassette in the IRs. As a result, we designed primers to amplify a DNA region extending from the end of the 5'-TR sequence of the IR gene (Supplementary Figure 7 and Supplementary Table 2: 5'-TR-amp primer, reporter-specific primer just upstream of the promoter) to a portion of the genomic sequence located less than 1kb downstream of the IR gene (Supplementary Figure 7 and Supplementary Table 2, genomic primers 1-20). The amplification products had a total length between 2kb and 3.9kb and spanned the following elements: 183bp of 5'-TR, promoter, EGFP cassette, promoter index, barcode, 3'-TR, and a small fraction of genomic DNA downstream (Supplementary Figure 7). We used this approach to check if the locus-specific genomic region downstream of the DSB remained associated with the correct promoter after Cas9 targeting. A match to a different promoter than expected would otherwise indicate a translocation.

We experienced major difficulties to obtain sufficient quality amplification product for sequencing (see section "Amplification issues" below). Moreover, four of the twenty DNA regions delivered amplification product, but the corresponding sequences did not match any reporter elements upstream or downstream of the expected DSB site, besides a short region surrounding the 5'-TR or genomic primer (Supplementary Table 1). This was likely caused by unspecific primer binding, since proper amplification of the targeted IR loci using the designed primers should at least contain the EGFP reporter sequence downstream of the expected DSB site. We were able to amplify and sequence five of the twenty IR loci. The promoter was correct for all five IRs with valid sequences (Supplementary Table 1). Although we cannot exclude the possibility of translocations, our data do not suggest that such events would be frequent nor that they would have had a major impact on our results and conclusions thereof.

**Genomic Primer**	**Chromosome**	**Base**	**Promoter**	**Strand**	**Amplification product**	**Sequence**	**Valid sequence (match IR downstream DSB)**	**Promoter match**
**1**	4	43734244	Oct4	+	no	NA	NA	NA
**2**	9	13630461	Hoxb1	-	yes	yes	**match**	**match**
**3**	20	146955246	Hoxb1	+	yes	no	NA	NA
**4**	6	37604358	CMV	-	yes	yes	no match	NA
**5**	14	96909472	Hoxb1	-	no	NA	NA	NA
**6**	9	115555263	Hoxb1	+	yes	yes	**match**	**match**
**7**	11	69347077	CMV	-	yes	yes	**match**	**match**
**8**	1	183806468	Hoxb1	-	yes	yes	no match	NA
**9**	12	4925222	Oct4	+	no	NA	NA	NA
**10**	6	6066083	Hoxb1	-	no	NA	NA	NA
**11**	6	26952426	CMV	-	yes	yes	**match**	**match**
**12**	2	171545278	Oct4	-	no	NA	NA	NA
**13**	14	101980700	Oct4	+	NA*	NA	NA	NA
**14**	4	108911304	PGK	-	yes	yes	no match	NA
**15**	6	15721098	Oct4	-	yes	yes	no match	NA
**16**	11	62252522	Oct4	+	no	NA	NA	NA
**17**	1	183049789	CMV	-	no	NA	NA	NA
**18**	6	86415855	Hoxb1	+	no	NA	NA	NA
**19**	16	97566570	cMyc	-	no	NA	NA	NA
**20**	9	22558808	Hoxb1	-	yes	yes	**match**	**match**

* No reliable primer could be designed.

Supplementary Table 1: Validation of promoter sequences for 20 randomly chosen IRs. Column "Genomic primer" denotes one of 20 primers targeting a genomic region downstream of the IR barcode, according to Supplementary Table 4. Columns "Chrom" to "Barcode" specify IR-related information, namely the chromosome, location within the chromosome, promoter, strand, and barcode. Column "Amplification product" specifies whether we were successful in obtaining an amplification product. Column "Sequence" indicates whether we could sequence the amplification product (NA when no amplification product was available). Column "Valid sequence" indicates "match" if the sequence corresponds to the intended IR locus, and "no match" otherwise (NA if no sequence was available). Column "Promoter match" indicates "match" if the promoter for a valid sequenced IR locus corresponds to the expected promoter, "no match" otherwise (NA if no valid sequence could be obtained).

The large IR DNA sequences were notoriously challenging to amplify, as expected. Amplification was further complicated by a majority of low-abundance IRs, since most IRs were only present in a fraction of the cells in the population. Moreover, by design we had to use a combination of locus-specific (genomic DNA) and reporter-specific (reporter DNA) primers so as to amplify the DNA at the desired location only when containing a reporter but also regardless of whether the region upstream of the expected DSB matched the expected one. This meant that at least one of the primers had to target one of the TRs, which was the only reporter element upstream of the promoter or downstream of the unique barcode. However, TR sequences are abundant in the genomic DNA of mES cells, making it difficult to design primers that would specifically recognize the intended sequence. As a result, unspecific primer binding led to unspecific and noisy sequence data. To mitigate unspecific binding, we also designed longer primers than usual (32bp).

Primer name	IR locus (chr. & base)		IR promoter	Promoter index	IR Barcode	IR strand	Primer sequence (5'-3')
**Genomic 1**	4	43734244	Oct4	GAGCG	TCTACATTTACGCATCGC	+	GTCAGAGGATATTAGAAAAGGCAGGAAGATAG
**Genomic 2**	9	13630461	Hoxb1	CGTCT	TAAGCAACTGATGCCGAC	-	CTCTTATATACATGGACTACGGTGGAGTAAAG
**Genomic 3**	20	146955246	Hoxb1	CGTCT	GATGTGTTTTGATGCCAC	+	GAGGTTAATTCTGGTAAGGGCTCAAAAGATG
**Genomic 4**	6	37604358	CMV	CAGCT	TCACCAGATTTCCGTTGG	-	CTACTCATTATTATTAGACCCCACCCCATCTG
**Genomic 5**	14	96909472	Hoxb1	CGTCT	TGACGCTTAAGCTACGAC	-	GTCTCAGGAACATTGGGAATTATACTTGACTG
**Genomic 6**	9	115555263	Hoxb1	CGTCT	TTCCCAACCTAAGGTTCC	+	TGAAAGATAATAAAGAACGGGCCTTGGTTG
**Genomic 7**	11	69347077	CMV	CAGCT	ATGCACAATGAGCGAAAC	-	GAGAGTGTAGATTAGTCAATTCAGGATCTG
**Genomic 8**	1	183806468	Hoxb1	CGTCT	TGTCTCCTAACGTGGTGT	-	ATATAAAGTTAGGACCTCTGTTCACTCCAGTC
**Genomic 9**	12	4925222	Oct4	GAGCG	CGATTTCAAATGTCCTTT	+	CGACACGTTTCTTTCTACATACTCCCTTTGTA
**Genomic 10**	6	6066083	Hoxb1	CGTCT	ACCGCGCTAAAAACATAT	-	GGATTTCTAATTCTATCCGTCCCTAACAAG
**Genomic 11**	6	26952426	CMV	CAGCT	CCTTTCGCGATAGCTCTG	-	TCTTCAGAAAATAGTCATACCCTCCTACACTG
**Genomic 12**	2	171545278	Oct4	GAGCG	AAAACTAGAGGAGAATAC	-	CTACAGTAGAGAGGCAGATACATGTTATTAGC
**Genomic 13**	14	101980700	Oct4	GAGCG	CTAATCGAGTAACATCAC	+	no sequence-specific primer
**Genomic 14**	4	108911304	PGK	TGTCT	CTTCCACCCAGCTACGGA	-	GATAGAACGTATCAATGTGGCTAAAACTCCCA
**Genomic 15**	6	15721098	Oct4	GAGCG	ACGAAATCGGGCCAGATC	-	TTATTTCATCTTCAATGGCACCTCATCTGC
**Genomic 16**	11	62252522	Oct4	GAGCG	AGGCCAGTGTATTTACGT	+	GCTCATCCTGCTCACCTGTTTACTATCTTAT
**Genomic 17**	1	183049789	CMV	CAGCT	AATGCCTCTAGCAGTAGG	-	GAGTTCCCAGAATAAACAGTCCAAGTCTTAAC
**Genomic 18**	6	86415855	Hoxb1	CGTCT	GATAAGGTAGCCAGAGAT	+	GATCTCTTCAAAACACACACACATTCCTCTG
**Genomic 19**	16	97566570	cMyc	ATACA	AGGGACTCCAGAAAGAAC	-	CCTTTCTCTGCCAGCTTTTAAACGTAATGAG
**Genomic 20**	9	22558808	Hoxb1	CGTCT	TTGCCCGACATCCCATTG	-	CTCTTTTAGCATTACACTCACGATCTGCAAAC
**5'-TR-amp**							GCGGTAAGTGTCACTGATTTTGAACTATAACG

Supplementary Table 2: Primers used to investigate the frequency of translocations upon Cas9 targeting. Listed are the sequences of the 20 IR-specific genomic primers, plus the sequence of the 5'-TR primer.

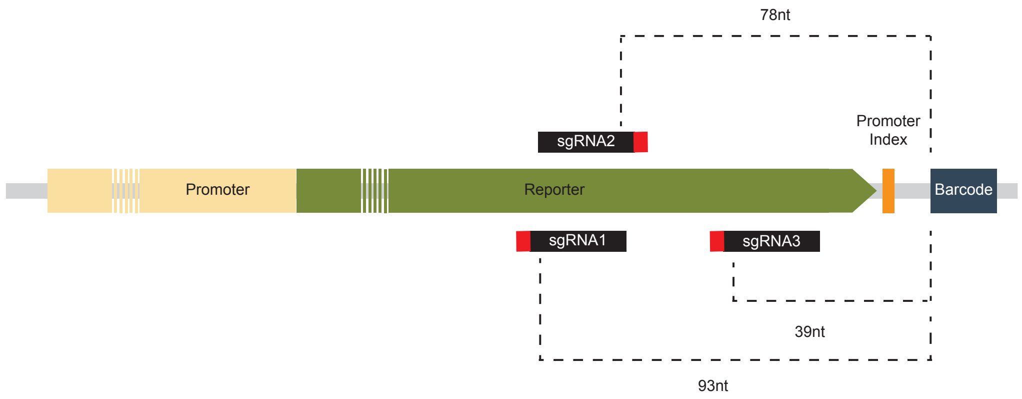

Supplementary Figure 8: Distance between the IR barcode and the sites targeted by sgRNA1-3 within the IR gene body.

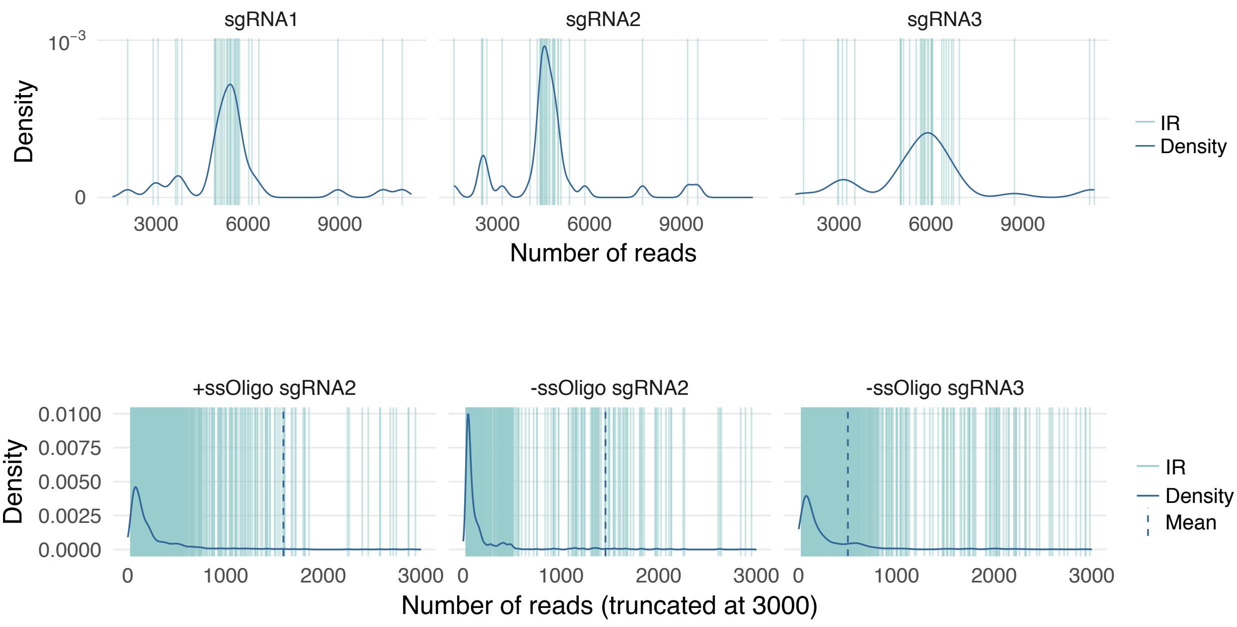

Supplementary Figure 9: Read coverage per IR in TRIP cell line and TRIP pool experiments. The average read coverage per IR was significantly larger in the TRIP cell line (top) than in the TRIP pool (bottom).

**Guide RNA / Oligo for HDR**	**Target sequence**	**Oligonucleotide sequence**
sgRNA1	GCCGAGAGTGATCCCGGCGG	CACCGCCGAGAGTGATCCCGGCGG<br>AAACCCGCCGGGATCACTCTCGGC
sgRNA2	CGCCGGGATCACTCTCGGCA	CACCgCGCCGGGATCACTCTCGGCA<br>AAACTGCCGAGAGTGATCCCGGCGc
sgRNA3	AGTGTCACCTAAATCGTATG	CACCgAGTGTCACCTAAATCGTATG<br>AAACCATACGATTTAGGTGACACTc
**Custom single-stranded oligonucleotide (ssODN)**	**Sequence**<br>tgagcaaagaccccaacgagaagcgcgatcacatggtcctgctggagttcgtgaccgcGATTACAGATATCATCAACAGcggcatggacgagctgtac aagtaagaattcgcggccgcatacgatttaggtgacactgcag	
**mCherry amplification primers**	**Primer sequence**	
**Fragment.FOR**	TGCTGGGGAGCGGCCGCTATTAATAGTAATCAATTACGGGGTCATTAGTTCATAGCCCA	
**Fragment.REV**	CGCATCAGGCGCCCCTGCAGGCCAGCTGGTTCTTTCCGCCTCAGAAG	

Supplementary Table 3: Sequences of the guide RNAs and the custom oligo for insertion by HDR used in the RNA-guided Cas9 targeting experiments of TRIP cell lines and pools. mCherry amplifications primers.

**Targeted cells**	**Primer name**	**Illumina sequencing adaptor**	**Index sequence**	**Complementary sequence**
TRIP cell line (MiSeq)	PB-cDNA-forward-1-BC-(1-4)	ACACTCTTTCCCTACACGACGCTCTTCCGATCT	NNNNNNNNNN	GTCACAAGGGC CGGCCACAA
TRIP cell line (MiSeq)	PB-cDNA-Reverse-5	ACGGCAACATCCTGGGGCACAAG		
TRIP cell pool (HiSeq)	PB-cDNA-Reverse-3	CGACAACCACTACCTGAGCACCCA		
TRIP cell pool (HiSeq)	Thio-Solexa-Ad-SE-1	AATGATACGGCGACCACCGAGATCT ACACTCTTTCCCTACACGACGCTCTTCCGAT*C *T		
TRIP cell pool (HiSeq)	PB-cDNA-Reverse-2a	CAAGCAGAAGACGGCATACGAGATGTGACTG GAGTTCAGACGTGTGCTCTTCCGATCTCTGAT CACATGGTCCTGCTGGAGTT		

Supplementary Table 4: Sequences of the primers used in preparation of the TRIP cell line and TRIP cell pool samples for sequencing following RNA-guided Cas9 targeting.

Primer name	Illumina sequencing primer	Index sequence	UID 16-nt	Annealing part of primer
**PB-cDNA-f-UID-16n-1**	ACACTCTTTCCCTACACGACGCTCTTCCGATCT	AACGTGAT	NNNNNNNNNNNNNNNN	GTCACAAGGGCCGGCCACAA
**PB-cDNA-f-UID-16n-2**	ACACTCTTTCCCTACACGACGCTCTTCCGATCT	AAACATCG	NNNNNNNNNNNNNNNN	GTCACAAGGGCCGGCCACAA
**PB-cDNA-f-UID-16n-3**	ACACTCTTTCCCTACACGACGCTCTTCCGATCT	ATGCCTAA	NNNNNNNNNNNNNNNN	GTCACAAGGGCCGGCCACAA
**PB-cDNA-f-UID-16n-4**	ACACTCTTTCCCTACACGACGCTCTTCCGATCT	AGTGGTCA	NNNNNNNNNNNNNNNN	GTCACAAGGGCCGGCCACAA
**PB-cDNA-f-UID-16n-5**	ACACTCTTTCCCTACACGACGCTCTTCCGATCT	ACCACTGT	NNNNNNNNNNNNNNNN	GTCACAAGGGCCGGCCACAA
**PB-cDNA-f-UID-16n-6**	ACACTCTTTCCCTACACGACGCTCTTCCGATCT	ACATTGGC	NNNNNNNNNNNNNNNN	GTCACAAGGGCCGGCCACAA
**PB-cDNA-f-UID-16n-7**	ACACTCTTTCCCTACACGACGCTCTTCCGATCT	CAGATCTG	NNNNNNNNNNNNNNNN	GTCACAAGGGCCGGCCACAA
**PB-cDNA-f-UID-16n-8**	ACACTCTTTCCCTACACGACGCTCTTCCGATCT	CATCAAGT	NNNNNNNNNNNNNNNN	GTCACAAGGGCCGGCCACAA
**PB-cDNA-f-UID-16n-9**	ACACTCTTTCCCTACACGACGCTCTTCCGATCT	CGCTGATC	NNNNNNNNNNNNNNNN	GTCACAAGGGCCGGCCACAA
**PB-cDNA-f-UID-16n-10**	ACACTCTTTCCCTACACGACGCTCTTCCGATCT	ACAAGCTA	NNNNNNNNNNNNNNNN	GTCACAAGGGCCGGCCACAA
**PB-cDNA-f-UID-16n-11**	ACACTCTTTCCCTACACGACGCTCTTCCGATCT	CTGTAGCC	NNNNNNNNNNNNNNNN	GTCACAAGGGCCGGCCACAA
**PB-cDNA-f-UID-16n-12**	ACACTCTTTCCCTACACGACGCTCTTCCGATCT	AGTACAAG	NNNNNNNNNNNNNNNN	GTCACAAGGGCCGGCCACAA

Supplementary Table 5: Sequences of the UID-specific primers used in preparation of the TRIP cell pool samples for sequencing following RNA-guided Cas9 targeting.

PCR amplification steps	Temperature	Time	
Assembly of the reaction	98˚C	03:00	1X
	98˚C	00:15	2X
Reagents:	65˚C	00:30	
PB-cDNA-Reverse-3 10µM	72˚C	01:00	
PB-cDNA-f-UID-16n-x 10µM	4˚C	Forever	1X
Exonuclease treatment	37˚C	01:00:00	1X
Reagent:	98˚C	05:00	1X
Exonuclease-I (20 U/µl) 3µl	4˚C	Forever	1X
Final amplification	98˚C	00:15	24X
Reagents:	65˚C	00:30	
Thio-Solexa-Ad-SE-1 10µM	72˚C	01:00	
PB-cDNA-Reverse-2a 10µM	72˚C	05:00	1X

Supplementary Table 6: PCR amplification protocol parameters.

Tested IR	Chromosome	Base	Strand	Barcode	Promoter	Index
1	4	43734244	+	TCTACATTTACGCATCGC	Oct4	GAGCG
2	9	13630461	-	TAAGCAACTGATGCCGAC	Hoxb1	CGTCT
3	20	146955246	+	GATGTGTTTTGATGCCAC	Hoxb1	CGTCT
4	6	37604358	-	TCACCAGATTTCCGTTGG	CMV	CAGCT
5	14	96909472	-	TGACGCTTAAGCTACGAC	Hoxb1	CGTCT

General reporter primers (5'->3')	**Primer sequence**
PB-Valid.3-Out primer-1	TCACATGGTCCTGCTGGAGTT
Inner-1	TCCTGCTGGAGTTCGTGACC
IR locus-specific genome spanning primers	**Primer sequence**
GEMP_mval_1_outer	TAAACCAGTCACTTTCTTGCACAGC
GEMP_mval_1_inner	TAAACCAGTCACTTTCTTGCACAGC
GEMP_mval_2_outer	ACTCCCAAATGCTTGCTCCAACTCT
GEMP_mval_2_inner	TCTATGCCCTCTTCCTCATCAGCTGT
GEMP_mval_3_outer	CAATGGGCACACACATGGGTTCAAG
GEMP_mval_3_inner	ATATGGAGAGAGAAGATGGAGTTCC
GEMP_mval_4_outer	AGGCAAGGGGTTTCATCACACACAG
GEMP_mval_4_inner	AGGAGAAGACTGGACATGATGGTGC
GEMP_mval_5_outer	TCGTGAAACTGGAAAGGCAAATGGT
GEMP_mval_5_inner	GCCACAAATGATATGGGCTGGAGGA
Sequencing primer (5'->3')	**Primer sequence**
PB-Valid.Gen.Seq-1	CCGGGATCACTCTCGGCA

Supplementary Table 7: List of five IRs from the TRIP pool that were randomly selected to validate the association between barcode and promoter index prior to Cas9 targeting. Sequences provided as a Source Data file.

Supplementary References:

Jiang *et al.* Induction of site-specific chromosomal translocations in embryonic stem cells by CRISPR/Cas9. *Sci. Rep.* **6,** 21918 (2016).
Lekomtsev *et al.* Efficient generation and reversion of chromosomal translocations using CRISPR/Cas technology. *BMC Genomics* **17,** 739 (2016).
