# Supplementary material for: Multiplexed Cas9 targeting reveals genomic location effects and gRNA-based staggered breaks influencing mutation efficiency
Source: Nat Commun. 2019 Apr 8;10:1598. doi: 10.1038/s41467-019-09551-w (PMC6453899; doi:10.1038/s41467-019-09551-w)
Supplement: Supplementary file 2 — Reporting Summary [file 41467_2019_9551_MOESM2_ESM.pdf]

nature research

Corresponding author(s): Lodewyk Wessels, Maarten van Lohuizen

Last updated by author(s): Mar 6, 2019

Reporting Summary

Nature Research wishes to improve the reproducibility of the work that we publish. This form provides structure for consistency and transparency in reporting. For further information on Nature Research policies, see Authors & Referees and the Editorial Policy Checklist.

Statistics

For all statistical analyses, confirm that the following items are present in the figure legend, table legend, main text, or Methods section.

n/a	Confirmed	
☐	☒	The exact sample size (*n*) for each experimental group/condition, given as a discrete number and unit of measurement
☒	☐	A statement on whether measurements were taken from distinct samples or whether the same sample was measured repeatedly
☐	☒	The statistical test(s) used AND whether they are one- or two-sided<br>*Only common tests should be described solely by name; describe more complex techniques in the Methods section.*
☐	☒	A description of all covariates tested
☐	☒	A description of any assumptions or corrections, such as tests of normality and adjustment for multiple comparisons
☐	☒	A full description of the statistical parameters including central tendency (e.g. means) or other basic estimates (e.g. regression coefficient) AND variation (e.g. standard deviation) or associated estimates of uncertainty (e.g. confidence intervals)
☐	☒	For null hypothesis testing, the test statistic (e.g. *F*, *t*, *r*) with confidence intervals, effect sizes, degrees of freedom and *P* value noted<br>*Give P values as exact values whenever suitable.*
☒	☐	For Bayesian analysis, information on the choice of priors and Markov chain Monte Carlo settings
☒	☐	For hierarchical and complex designs, identification of the appropriate level for tests and full reporting of outcomes
☐	☒	Estimates of effect sizes (e.g. Cohen's *d*, Pearson's *r*), indicating how they were calculated

Our web collection on statistics for biologists contains articles on many of the points above.

Software and code

Policy information about availability of computer code

Data collection	UNIX command-line tools, SRA-toolkit version 2.9.0 for retrieving and manipulating HCoDES sequence data from SRA, fastQC version 0.11.4 to assess the quality of sequence data.
Data analysis	R Bioconductor package ShortRead to identify sequences such as reporter barcodes, BowTie version 2.0 to align reads to the mouse genome, NCBI BLAST 2.7.1+ to align HCoDES sequences to the reference HCoDES sequence, numerous statistical analysis and plotting functions from the base R software version 3.2.3 (such as lm, anova, cor.test, ...) as well as the following additional R packages: reshape2_1.4.2, ggbeeswarm_0.6.0, heplots_1.3-4, gridExtra_2.3, viridis_0.4.0, viridisLite_0.2.0, ggsci_2.8, ggthemes_3.4.0, ggplot2_2.2.1, data.table_1.10.4-3, dplyr_0.7.4, Gviz_1.14.7, GenomicRanges_1.22.4, GenomeInfoDb_1.6.3, IRanges_2.4.8, car_2.1-6, stringr_1.2.0.

For manuscripts utilizing custom algorithms or software that are central to the research but not yet described in published literature, software must be made available to editors/reviewers. We strongly encourage code deposition in a community repository (e.g. GitHub). See the Nature Research guidelines for submitting code & software for further information.

Data

Policy information about availability of data

All manuscripts must include a data availability statement. This statement should provide the following information, where applicable:

- Accession codes, unique identifiers, or web links for publicly available datasets
- A list of figures that have associated raw data
- A description of any restrictions on data availability

High-throughput sequence data and accompanying processed data that support the findings of this study have been deposited in GEO with accession code GSE127752.

Field-specific reporting

Please select the one below that is the best fit for your research. If you are not sure, read the appropriate sections before making your selection.

☒ Life sciences ☐ Behavioural & social sciences ☐ Ecological, evolutionary & environmental sciences

For a reference copy of the document with all sections, see nature.com/documents/nr-reporting-summary-flat.pdf

Life sciences study design

All studies must disclose on these points even when the disclosure is negative.

Sample size	The cell lines contained 36 reporter integrations, the cell pools contained several thousands of reporter integrations.
Data exclusions	We excluded barcoded reporters which could not be mapped against the mouse genome, had very low sequence coverage (count below 30) or whose barcode could not be uniquely identified. The reason for the exclusion was to guarantee the reliability and robustness of subsequent statistical analyses.
Replication	Experiments to obtain mapping, normalization and expression reads were performed in duplicate. We also performed 12 replicates per guide RNA-Cas9 targeting combination of TRIP pools. The data assessed in our study (mutation frequencies and patterns) were reproducible, since they were highly correlated across the different replicates.
Randomization	Reporter genes had identical sequence, were assigned random promoter sequences, and were randomly integrated into the genomes of mouse embryonic stem cells.
Blinding	The integration of barcoded reporters into the genomes was inherently random.

Reporting for specific materials, systems and methods

We require information from authors about some types of materials, experimental systems and methods used in many studies. Here, indicate whether each material, system or method listed is relevant to your study. If you are not sure if a list item applies to your research, read the appropriate section before selecting a response.

Materials & experimental systems

n/a	Involved in the study	
☒	☐	Antibodies
☐	☒	Eukaryotic cell lines
☒	☐	Palaeontology
☒	☐	Animals and other organisms
☒	☐	Human research participants
☒	☐	Clinical data

Methods

n/a	Involved in the study	
☒	☐	ChIP-seq
☒	☐	Flow cytometry
☒	☐	MRI-based neuroimaging

Eukaryotic cell lines

Policy information about cell lines

Cell line source(s)	The EStTA cell lines were established in-house. The single-promoter TRIP cell lines and multi-promoter TRIP pools were established in-house.
Authentication	Morphology assessment by microscopy. Growth rate assessment.
Mycoplasma contamination	All cell lines tested negative for mycoplasma contamination.
Commonly misidentified lines (See ICLAC register)	*Name any commonly misidentified cell lines used in the study and provide a rationale for their use.*
